# COVID-19: the challenges of transitioning a hands-on and interactive Honors Reproduction course to an online format

**DOI:** 10.1093/tas/txaa221

**Published:** 2020-12-02

**Authors:** Ky G Pohler, Rebecca K Poole, Gabriela D Melo

**Affiliations:** Pregnancy and Developmental Programming Area of Excellence, Department of Animal Science, Texas A&M University, College Station, TX

The COVID-19 pandemic has undoubtedly affected education systems through the closure of schools and universities. Like many universities, Texas A&M University also opted to transition to an online format, a task which is challenging for any institution. With the number of courses that were taught online pre-COVID, this varied not only by institution, but also by curriculum. For example, in the Fall 2019 semester pre-COVID, the Department of Animal Science at Texas A&M University offered approximately 94% of its undergraduate courses and 100% of its graduate courses in the traditional face-to-face format (this is excluding nontraditional courses; i.e., internships, research, etc.). Similar to other Animal Science departments, core curriculum courses include Animal Nutrition, Genetics, and Reproduction. One unique feature of Texas A&M’s Animal Science curriculum is that there is a separate Reproduction in Farm Animals (ANSC 333) course available only to eligible students in the Honors Program. This course is offered in the spring and fall semesters to undergraduate Animal Science Honors students and is designed to cover the physiological principles of reproductive processes in farm species. Traditionally, the classes are given in a face-to-face lecture format, in which twice a week the students are presented with fundamental concepts about the physiology and endocrinology of reproduction in farm animals. In addition, the students participate in a laboratory session once a week where they can review the learned content through practical and hands-on activities. Given that this course was traditionally taught face-to-face, as was the overwhelming majority of the undergraduate Animal Science curriculum, the aim of this paper was to share our experiences in transitioning this interactive Honors course to an online format.

## HONORS ANSC 333: BEFORE THE COVID-19 PANDEMIC

For the Honors Reproduction in Farm Animals course, the learning outcomes range from implementation of animal reproductive management strategies to effective communication skills, and these outcomes are assessed by different means. Every other week, the students receive a quiz covering the main contents of previous lectures, following the principles of formative assessment (Prégent, 1994). Through this method, both students’ and instructors’ benefit, as the student’s learning is appraised before any shortfalls are reflected in their exam grades (Nilson, 2010). In 5-wk intervals, the students take a more extensive exam, designed to assess their ability to apply the principles of reproductive biology toward improving reproductive efficiency of livestock species. In the laboratory sessions, discussion questions are presented to help students integrate information and connect basic concepts with real-world problems. Students are divided into groups of three or four to interactively discuss their ideas and subsequently defend their ideas to the class. Writing assignments are also part of the student’s activities, in which they are encouraged to use their educated opinions to answer a topic related to animal reproduction. Finally, at the end of the semester, there is a practical lab exam, when the students can demonstrate what they learned in the various laboratory sessions throughout the semester. Both the lecture and lab activities are accounted to evaluate students’ performance, as a summative assessment (Nilson, 2010).

As previously mentioned, this course is part of the Honors Program and is designed for academically outstanding students. From this perspective, the coursework is broad and goes beyond sharing essential content through lectures and assignments. The students are constantly encouraged to use their knowledge to foster critical thinking and utilize problem-solving skills. In this class, they also develop their scientific writing competence, learn how to interpret information displayed via multiple graphic forms of communication, and improve their listening and professional skills. By the end of the semester, the students are expected to have accumulated an extensive background to use in their scientific and professional careers as future leaders in agriculture.

## HONORS ANSC 333: DURING THE COVID-19 PANDEMIC

In the spring semester of 2020, the course was administered by various instructors including an assistant professor (Dr Ky Pohler), a postdoctoral research associate (Dr Rebecca Poole) and a PhD student (Gabriela D. Melo). The course had ten students enrolled with distinct backgrounds and selected by their outstanding academic performances. The start of the semester was January 8 and proceeded as usual until mid-March, when all face-to-face classes were temporarily cancelled due to the coronavirus outbreak. During this time, the faculty, staff, and student leaders were given 1 wk to coordinate and plan the remainder of the semester by transitioning all face-to-face classes to an online format. This was a daunting task and a collective effort was needed to continue to maintain positive interactions between students and professors. In addition, there was a great concern surrounding the student’s accessibility to remote education, and how this would affect their learning process. To aide in this transition for instructors and students, Texas A&M created the “Keep Teaching” (keepteaching.tamu.edu) and “Keep Learning” (keeplearning.tamu.edu) initiative to provide support. These resources provide numerous guides and examples to help create a consistent and high-quality learning environment.

For the Reproduction in Farm Animals course, one of the main priorities was to ensure that all the topics would still be covered. For this, all the lectures were recorded through Zoom software (Zoom Video Communications, San Jose, CA) while the screen was being shared. The videos were uploaded to the university’s portal, where all the students could have access to the lectures at any desired time. This asynchronous approach was ideal for students in rural/remote areas where live-streaming can be difficult and ensured that they could have access and download all course material. The designated course time was instead used for the students and instructor to discuss the course material and clarify any questions or concerns via Zoom. Minimizing stressful situations should be a priority for instructors as it can adversely affect learning (Whitman et al., 1986), and this was a huge concern during and after the transition phase. Fortunately, the online course approach was favorable among students: “Dr. Pohler truly cared that we understood and discussed the material in the class. He was very understanding during this hard time with school transitioning online. I appreciated his compassion.” Moreover, the use of visual aids have a great impact on how people retain information (Poffenbarger, 2017), therefore to enhance students’ comprehension of the material remotely, an iPad drawing tool was used by the instructor during the meetings to draw on diagrams to emphasize and answer various questions. The students had the option to use their cameras or not, so they would feel comfortable. To guarantee accessibility to all the students, the virtual meetings were recorded and posted in the portal.

The laboratory activities demand the use of specialized facilities as well as hands-on activities with animals and various reproductive equipment. Unfortunately, due to the short transition period and reduced staff, many laboratory activities could not be adequately recorded. Instead, the theoretical material was reformulated to meet the students’ needs. The content was presented in more detail, with additional practical advice and comments that would typically be emphasized during the laboratory session. The students also had access to good quality images and videos each with a detailed explanation. The material was uploaded to the university’s portal and was available for the remainder of the semester. Like the lecture format, the designated laboratory session time was also used to discuss the material and clarify any questions via Zoom.

Transitioning on-site quizzes, discussions, writing assignments, and exams to an online format required some creativity and flexibility to maintain the same evaluation criteria of the curriculum. Quizzes were uploaded to the university’s portal, the students were notified and had 24 h to answer the questions. This approach was done to ensure all students would have the opportunity to respond. The 1-h laboratory session via Zoom was destined to discuss and debate the discussion questions and writing assignments, and the students’ participation was encouraged and taken into consideration when evaluating their progress. For both the discussion questions and writing assignments, students made their responses available on the portal. Surprisingly, the students were more prone to participate via Zoom and the discussions were productive and well-developed by the students. The exams were conducted through Zoom in breakout sessions containing three to four participants per breakout room. The students were given the exam questions and allowed to discuss within their group while the instructor transited through each breakout room to clarify any answers and at times even enrich some of the discussions. After finishing the exam, the students had to individually upload their answers to the portal. Unfortunately, it was not possible to assess the practical skills typically acquired by the students in the laboratory sessions. One solution was to adapt the lab final to an assignment where the students could draw or schematize one of the reproductive processes learned during the course. This helps with retention of information and intellectual engagement with the material when compared to conventionally typed answers (Mueller et al., 2014).

Overall, the transition process was challenging for both instructors and students; however, the students responded positively to the transition and ultimately felt comfortable participating in class activities such as leading discussions. To encourage students to think critically, the quizzes and exams were reformulated with questions whose answers could not be so easily found in books or online articles. This adjustment was well-accepted, and positively reflected in their grades and evaluation of the course: “Dr. Pohler has a passion for reproduction, which I think really made the course interesting. He was always available for questions, and always related the information in class to world examples, which I think was the best part of the course. I am glad I took this course with Dr. Pohler because I feel I was able to learn a lot instead of fretting over the grade I would receive. He wanted his students to succeed and understand the information.” When comparing grade outcomes of this course from the previous two semesters prior to COVID-19 and to this cohort, there was no difference. However, it will be interesting to observe if this trend continues since this group of students attended half the semester in person.

## HONORS ANSC 333: MOVING FORWARD

For the fall 2020 semester, it was decided that this course will not return to a face-to-face format, which allowed for further refinement and improvement to the online teaching model. Given the positive feedback from students in the previous semester, the blended learning approach with components of asynchronous (i.e., Canvas) and synchronous (i.e., Zoom) teaching was utilized for the fall 2020 semester. Even before the COVID-19 pandemic, blended learning approaches have been shown to be very successful because they provide both teacher-directed instruction (i.e., synchronous via face-to-face or Zoom) as well as allowing the students a degree of control specifically regarding the time and pace at which they can watch lecture recordings (i.e., asynchronous via prerecorded lectures; Wengreen et al., 2015). This approach seems to even be beneficial for the students that fully take advantage of it. For example, while there is no difference in the overall course grade between the students who regularly attend the optional synchronous Zoom sessions and those that do not (87% vs. 86%, respectively), the students who do regularly attend improved their second exam score by on average seven points when compared to the first exam. Whereas, the students who do not regularly attend saw on average a two-point reduction between the first and second exam. Regardless if the course is presented in the traditional face-to-face or online format, the instructors have an obligation to facilitate learning to its greatest potential and ultimately prepare the students to be future leaders in the field of Animal Science.
